# Targeting Toll-like receptor 4 with CLI-095 (TAK-242) enhances the antimetastatic effect of the estrogen receptor antagonist fulvestrant on non-small cell lung cancer

**DOI:** 10.1007/s12094-020-02353-3

**Published:** 2020-05-04

**Authors:** S. Fan, Y. Liao, W. Qiu, L. Li, D. Li, X. Cao, B. Ai

**Affiliations:** 1 Department of Thoracic Surgery, The First Affiliated Hospital of Xiamen University, Xiamen University, Xiamen, 361000 Fujian Province China; 2 Department of Thoracic Surgery, Union Hospital, Tongji Medical College, Huazhong University of Science and Technology, Jiefang Dadao Street 1277, Wuhan, 430030 Hubei Province China; 3 Department of Thoracic Surgery, Tongji Hospital, Tongji Medical College, Huazhong University of Science and Technology, Wuhan, 430030 Hubei Province China

**Keywords:** Fulvestrant, Antimetastasis, CLI-095 (TAK-242), NSCLC, Antagonist

## Abstract

**Purpose:**

Estrogen plays a critical role in the invasiveness and metastasis of non-small cell lung cancer (NSCLC) through estrogen receptor β (ERβ). However, the antimetastatic effect of the ERβ antagonist fulvestrant was still limited in NSCLC patients. Recently, Toll-like receptor 4 (TLR4) signaling was implicated in NSCLC metastasis. Our present study aimed to evaluate the synergistic antimetastatic effect of a combination of fulvestrant and the TLR4-specific inhibitor CLI-095 (TAK-242) on human NSCLC cells.

**Methods:**

The expression levels of ERβ and TLR4 were detected by immunohistochemical (IHC) analysis of 180 primary NSCLC and 30 corresponding metastatic lymph node samples. The association between ERβ and TLR4 expression was analyzed. The aggressiveness of NSCLC cells treated with fulvestrant, CLI-095 or the drug combination and formation status of their invadopodia, invasion-associated structures, were investigated. The protein levels in NSCLC cells in different groups were determined by Western blot and immunofluorescence analyses.

**Results:**

Here, a positive correlation between ERβ and TLR4 expression was observed in both primary NSCLC tissue (Spearman’s Rho correlation coefficient = 0.411, *p* < 0.001) and metastatic lymph node tissue (Spearman’s Rho correlation coefficient = 0.374, *p* = 0.009). The protein levels of ERβ in NSCLC cell lines were decreased by fulvestrant, and this suppressive effect was significantly enhanced when fulvestrant was combined with CLI-095 (*p* < 0.05). Both the migration and invasion of NSCLC cells were suppressed by fulvestrant or CLI-095 alone, and the combination of fulvestrant + CLI-095 showed the strongest inhibitory effect (*p* < 0.05). In addition, the results demonstrated that CLI-095 also helped fulvestrant restrict the formation and function of invadopodia in NSCLC cells (*p* < 0.05).

**Conclusions:**

Collectively, our study results suggested that CLI-095 enhances the antimetastatic effect of fulvestrant on NSCLC and provided support for further investigation of the antitumor activity of combined therapy with antiestrogen and anti-TLR4 agents in the clinic.

**Electronic supplementary material:**

The online version of this article (10.1007/s12094-020-02353-3) contains supplementary material, which is available to authorized users.

## Introduction

17-β-Estradiol (E2) has been known for decades as the primary reproductive hormone, as it is the major and most potent product synthesized by the ovaries in premenopausal females [1, 2]. Since estrogen can also be synthesized by the placental syncytiotrophoblast, adipose tissue, skin fibroblasts, the bone, and the brain in both sexes, estrogen is known to play crucial physiological roles in several nonproductive systems, such as the cardiovascular, neuronal and skeletal systems [3–5]. However, a recent Women’s Health Initiative (WHI) study conducted with over 16,000 postmenopausal women receiving estrogens for daily hormone replacement therapy (HRT) revealed an increasing risk of lung cancer mortality in these women compared to women receiving a placebo treatment for 5 years [6]. Clinical trials indicated that estrogen exposure is a risk factor for lung carcinogenesis. Studies have continued to provide evidence that estrogen receptors (ERs), mainly subtype ERβ, are consistently expressed in lung cancer tissues and adenocarcinoma cell lines [7–9] and that high expression of ERβ predicts a very poor prognosis in lung cancer patients [7, 10–12]. Consistently, our previous studies first detected a significantly higher expression level of ERβ in metastatic lymph nodes of non-small cell lung cancer (NSCLC) patients than in primary tumor tissue. The results further indicated that treatment with estrogen or ERβ activation also significantly promotes lung cancer cell metastasis by upregulating invasiveness-associated matrix metalloprotease 2 (MMP2) both in vitro and in vivo [13]. Therefore, to markedly depress NSCLC metastasis and to finally detect a new treatment strategy for advanced lung cancer patients, inhibiting the effect induced by estrogen on NSCLC cells is essential.

Antiestrogens demonstrate impressive efficacy in the suppression of tumor progression and metastasis in the context of breast cancer [2, 3]. Accumulating evidence now supports a potential impactful role for antiestrogen reagents in inhibiting NSCLC development through ERβ. Data from urethane-induced primary NSCLC mouse models indicate that the inhibition of ERβ by fulvestrant can significantly reduce lung cancer formation and pleural metastasis [14]. In NSCLC cell lines, proliferation-associated IGF-1 and invasion-associated MMP2 expression levels are downregulated in response to antiestrogens in vitro [13, 15]. A clinical study including 26 women with second primary lung cancer among 6361 diagnosed breast cancer patients provided evidence of a longer cancer-specific survival rate in the patients treated with antiestrogens for breast cancer compared with those treated without antiestrogens [16]. In current clinical practice, a phase I study showed the safety and potential antitumor activity of the combined use of an epidermal growth factor receptor tyrosine kinase inhibitor (EGFR-TKI) and fulvestrant in postmenopausal women [17]. Nonetheless, no significant difference in the progression-free survival rate was detected in another phase II clinical study, which aimed to evaluate whether the addition of fulvestrant enhances the antitumor efficacy of erlotinib (a different EGFR-TKI) [18]. The results above suggest that it is still uncertain whether NSCLC patients can benefit from fulvestrant treatment. They also reveal that the understanding of antiestrogen theory remains incomplete and that a potential mechanism should be determined to enhance the antimetastatic effect of antiestrogen treatment strategies.

Toll-like receptor 4 (TLR4) is known as an important receptor in innate immunity and the first line of host defense [19]. The agonist that triggers TLR4 signaling, lipopolysaccharide (LPS), has been proven to be a major component of the outer membrane of Gram-negative bacteria [20]. Recent studies have provided evidence that TLR4 activation augments NSCLC cell adhesion to murine hepatic sinusoids and the formation of hepatic metastases in vivo, and an upregulation of MMP2 expression in response to LPS induction has been identified [21]. Other studies highlighted that TLR-4 increased the rate of lung metastasis in inflamed mice through the upregulation of C–C chemokine receptor type 2 (CCR2) expression [22] and proved that the TLR4/Myd88/NF-κB/MMP2 axis plays a crucial role in cancer metastasis [23, 24]. CLI-095, also known as TAK-242, is a novel cyclohexene derivative that specifically suppresses TLR4 signaling [25]. Although novel inhibition of NSCLC cell proliferation induced by knocking down TLR4 expression has been detected in vitro [26], the antimetastatic effect of specific TLR4 signaling inhibition is still unclear.

For this study, immunohistochemistry was used to detect the expression of ERβ and TLR4 in 180 samples of primary NSCLC tissue and 30 corresponding metastatic lymph node samples, and the relationship between these molecules was analyzed. Then, we used a combination of fulvestrant and CLI-095 to test the hypothesis that inhibiting TLR4 will enhance the antimetastatic effect of fulvestrant by suppressing NSCLC cell migration, invasion and metastasis. Protein expression levels in different groups were detected via Western blot and immunofluorescence analyses. In addition, we also analyzed the duration of formation of the key structure of cell metastasis, the invadopodia, by a 3D spheroid invasion assay and fluorescent gelatin degradation assay. Our results helped us to determine the negative effect of the combination of fulvestrant and CLI-095 on NSCLC metastasis progression and to provide evidence for a new strategy of advanced NSCLC patient treatment.

## Materials and methods

### Human tissue samples

Tissue samples were obtained from 180 confirmed NSCLC tumors and 30 corresponding metastatic lymph nodes resected between December 2013 and September 2015 and assessed in The Department of Pathology, Affiliated Tongji Hospital of Huazhong University of Science and Technology Tongji Medical College (Wuhan, China). The samples were obtained from 127 males and 53 females (mean age, 50.3 ± 4.7 years; range 21–77 years). Clinicopathological parameters, including age, sex, smoking index, pathological diagnosis and clinical stage, were obtained from the Tongji hospital records. The histological characterization and clinical–pathological staging of the samples was determined according to World Health Organization criteria [13]. Paraffin-embedded tumor specimens were used to create tissue microarray (Outdo Biotech Co., Ltd., Shanghai, China) blocks with 2-mm diameter cores for immunohistochemical (IHC) staining. Two tissue cores were obtained from each patient. The baseline characteristics of the patients are shown in Table 1. Informed consent was obtained before surgery. All patients involved consented to participate under the Clinical Patient ethical statement. Study approval for the study was obtained from the Research Ethics Committee of Tongji Medical College, Huazhong University of Science and Technology (IRB ID number 20141101).

**Table 1:** Correlation between ERβ and TLR4 expression and clinicopathological parameters in 180 cases of primary non-small cell lung carcinoma

	ERβ expression *N* (%)		*χ*^2^	*p* value	TLR4 expression *N* (%)		*χ*^2^	*p* value
	+	−			+	−		
*Age*								
Below median	78 (43.4)	28 (15.6)	0.008	0.927	86 (47.8)	20 (11.1)	0.207	0.650
Above median	54 (30.0)	20 (11.1)			58 (32.2)	16 (8.9)		
*Gender*								
Male	91 (50.6)	36 (20.0)	0.622	0.430	102 (56.7)	25 (13.9)	0.027	0.870
Female	41 (22.8)	12 (6.7)			42 (23.3)	11 (6.1)		
*Smoking index*								
< 400	84 (46.7)	35 (19.4)	1.353	0.245	90 (50.0)	29 (16.1)	0.937	0.333
≥ 400	48 (26.7)	13 (7.2)			50 (27.8)	11 (6.1)		
*Histological type*								
Squamous cell	36 (20.0)	14 (7.8)	0.063	0.802	44 (24.4)	6 (3.3)	2.769	0.096
Adenocarcinoma	96 (53.3)	34 (18.9)			100 (55.6)	30 (16.7)		
*T stage*								
T1–T2	112 (62.2)	36 (20.0)	2.336	0.126	119 (66.1)	29 (16.1)	0.086	0.770
T3–T4	20 (11.1)	12 (6.7)			25 (13.9)	7 (3.9)		
*N stage*								
N0	75 (41.7)	30 (16.7)	0.468	0.494	82 (45.6)	23 (12.8)	0.571	0.450
N1–3	57 (31.6)	18 (10.0)			62 (34.4)	13 (7.2)		
*Clinical stage*								
I–II	93 (51.7)	30 (16.7)	1.029	0.310	101 (56.1)	22 (12.2)	1.085	0.298
III–IV	39 (21.7)	18 (10.0)			43 (23.9)	14 (7.8)		

Details of the 30 metastatic lymph node samples with IIA-IIIB NSCLC and lymph node metastasis were provided in our previous study [13]. Patients with lymphadenitis or primary malignancies of the lymph node were excluded. Palliative care or surgical biopsy following informed consent was administered to 32 patients with inoperable Stage IIIb-IV primary NSCLC.

### Immunohistochemistry

Immunohistochemical staining was carried out using the avidin–biotin peroxidase method [13, 15]. All sections were deparaffinized in xylene, rehydrated in alcohol, incubated in hydrogen peroxide, blocked in 10% goat serum and then incubated overnight in 0.3% H_2_O_2_ at room temperature with primary antibodies (rabbit anti-human TLR4 polyclonal antibodies, 1:100, Abcam, Cat: ab13556 and rabbit anti-human ERβ monoclonal antibodies, 1:100, Abcam, Cat: ab3577), Immunoreactivity scores of the cancer tissue samples were determined based on the staining intensity and positive staining area according to the method described in Tang et al. [14]. A staining index (values 0–16), obtained as the intensity of positive staining [negative (1 scores), weak (2 scores), moderate (3 scores), or strong (4 scores)] and the proportion of immune-staining positive cells of interest [< 25% (1 scores), 25–50% (2 scores), 50–75% (3 scores), ≥ 75% (4 scores)] were calculated. A score of 1–16 was obtained by multiplying the staining intensity and positive cell proportion. A total score > 12 was defined as high expression, a score ≤ 8 was defined as low expression, and a score ≤ 4 was defined as negative expression.

### Cell culture and associated reagents

The human NSCLC cell lines A549 and H1793 were purchased from American Type Culture Collection (ATCC, Manassas, VA, USA). The culture condition was described in previous study [13] and were maintained in RPMI 1640(A549) or DMEM/F-12(H1793) supplemented with 10% fetal bovine serum (FBS). The NSCLC cells were treated with E2 (Sigma-Aldrich, St. Louis, MO, USA), fulvestrant, also known as Faslodex or ICI-182780 (7α-[9-[(4,4,5,5,5-pentafluoropentyl)-sulfinyl]nonyl]estra-1,3,5(10)-triene-3,17β-diol, Cayman Chemical), and CLI-095, also known as TAK242 (ethyl(6R)-6-[N-(2-chloro-4- fluorophenyl)sulfamoyl]cyclohex-1-ene-1-carboxylate, InvivoGen, USA) either alone or in combination. The doses of these drugs used in vitro and in vivo were comparable to previously described doses [13, 15, 27]. Each group of cells was treated for 48 h and harvested for further analysis. Cell culture experiments were performed using reagents formulated in 100% DMSO.

### Western blot analyses and Immunofluorescence

Western blot analyses and Immunofluorescence were performed as described by us previously [13, 27]. Briefly, total protein extract for each cell line was dissolved in lysis buffer and equal amounts of protein (40 μg) were analyzed by immunoblotting. The Immunofluorescence coverslips were observed under a fluorescence microscope (Olympus, Tokyo, Japan).

The primary antibodies used for the Western blot analyses and Immunofluorescence included rabbit anti-human ERβ (1:1,000) from Abcam (Cat: ab3577), rabbit anti-human TLR4 (1:1,000) from Abcam (Cat: ab13556), rabbit anti-human myd88 (1:1,000) from Proteintech (Cat: 23230-1-AP), and mouse anti-human GAPDH (1:10,000) from Cell Signaling Technology (CST; Cat: 51332; USA).

### Wound-healing assay

NSCLC cells were seeded in six-well plates for 24 h. After growth to confluence, the surface of the plate was scraped with a 200 μL pipette tip to generate a cell-free zone, and then incubated with RMPI 1640 containing 10% FBS for 24 h. Cells were photographed using a phase-contrast microscope (100×) as previously described [13].

### In vitro Transwell^®^ migration and invasion assays

Transwell migration and invasion assays were performed as described by us previously [13, 27]. Transwell^®^ Permeable Supports (24-well supports, inserts were 6.5 mm in diameter) (Corning, NY, USA) were used. In brief, NSCLC cells suspended in serum-free medium were added to the upper chamber at various densities depending on the cell line. Invasion assays were evaluated based on the number of cells invading a Transwell^®^ membrane coated with Matrigel^®^ (BD Biosciences, Bedford, MA, USA), and counting was conducted using an Olympus microscope (Olympus, Tokyo, Japan) at 100× magnification. Four fields were randomly selected for analysis. Detailed procedures are described elsewhere.

Migration assays were performed in 24‑well Transwell^®^ chambers containing polycarbonate filters with 8‑μm pores without Matrigel^®^. The remaining steps were identical to those described for the Transwell^®^ invasion assays.

### 3D spheroid invasion assay

The Cultrex^®^ 3D Spheroid Cell Invasion Assay Kit (Catalog: 3500-096-K, Trevigen, Gaithersburg, MD) was utilized for this procedure. A total of 1 × 10^5^ cells in 500 mL of medium containing 2.5% Matrigel and 5 ng/mL Spheroid Formation ECM were plated in 24-well plates coated with a collagen/Matrigel mixture. Spheres with protrusions were considered positive for cell invasion. Distances between the invasive cell frontier and spheroid edge were measured at 0, 72 and 144 h using an Olympus IX70 inverted microscope (Olympus, Tokyo, Japan). Each experiment was repeated twice, and each procedure was performed in triplicate.

### Fluorescent gelatin degradation assay

A QCM™ Gelatin Invadopodia Assay kit (Green) (Catalog: No. ECM670, Millipore, USA) was utilized for this procedure. Coverslips were cleaned with 20% nitric acid and coated with poly-L-lysine in a 24-well plate. Poly-L-lysine was fixed with 0.5% glutaraldehyde before adding FITC-conjugated gelatin. A thin layer of FITC-conjugated gelatin was applied to the coverslips and crosslinked using glutaraldehyde on ice for 10 min. Crosslinking was continued at room temperature for an additional 30 min. The coverslips were rinsed with PBS, incubated with 5 mg/mL sodium borohydride at room temperature for 3 min, rinsed again with PBS, incubated with 70% EtOH for 10 min, and dried at 37 °C for 15 min in a CO2 incubator. One hour before plating cells, the coverslips were quenched with RPMI-1640 medium containing 10% FBS at 37 °C. Cells were plated on the FITC-conjugated gelatin-coated coverslips and cultured in RPMI-1640 medium for 24 h to quantify the formation of invadopodia. Images were visualized by confocal microscopy (Olympus, Tokyo, Japan).

### Statistical analysis

Statistical analysis was performed using SPSS 19.0 statistical software (IBM, New York City, USA). The *χ*^2^ test was performed to analyze the correlations among ERβ and TLR4 expression and clinicopathological parameters, and Spearman’s rank correlation was used to analyze the correlations between ERβ and TLR4 expression in primary NSCLC and metastatic lymph node tissue samples. Comparisons between groups were analyzed using an unpaired *t* test. Data are presented as the mean ± SE. The remaining data are presented as the mean ± SD. *p* values < 0.05 were considered significant. All statistical analyses were performed using GraphPad Prism (version 7.00, GraphPad, San Diego, CA, USA).

## Results

### The relation between ERβ and TLR4 expression in primary NSCLC tumor tissue samples and metastatic lymph node samples

**Fig. 1:**
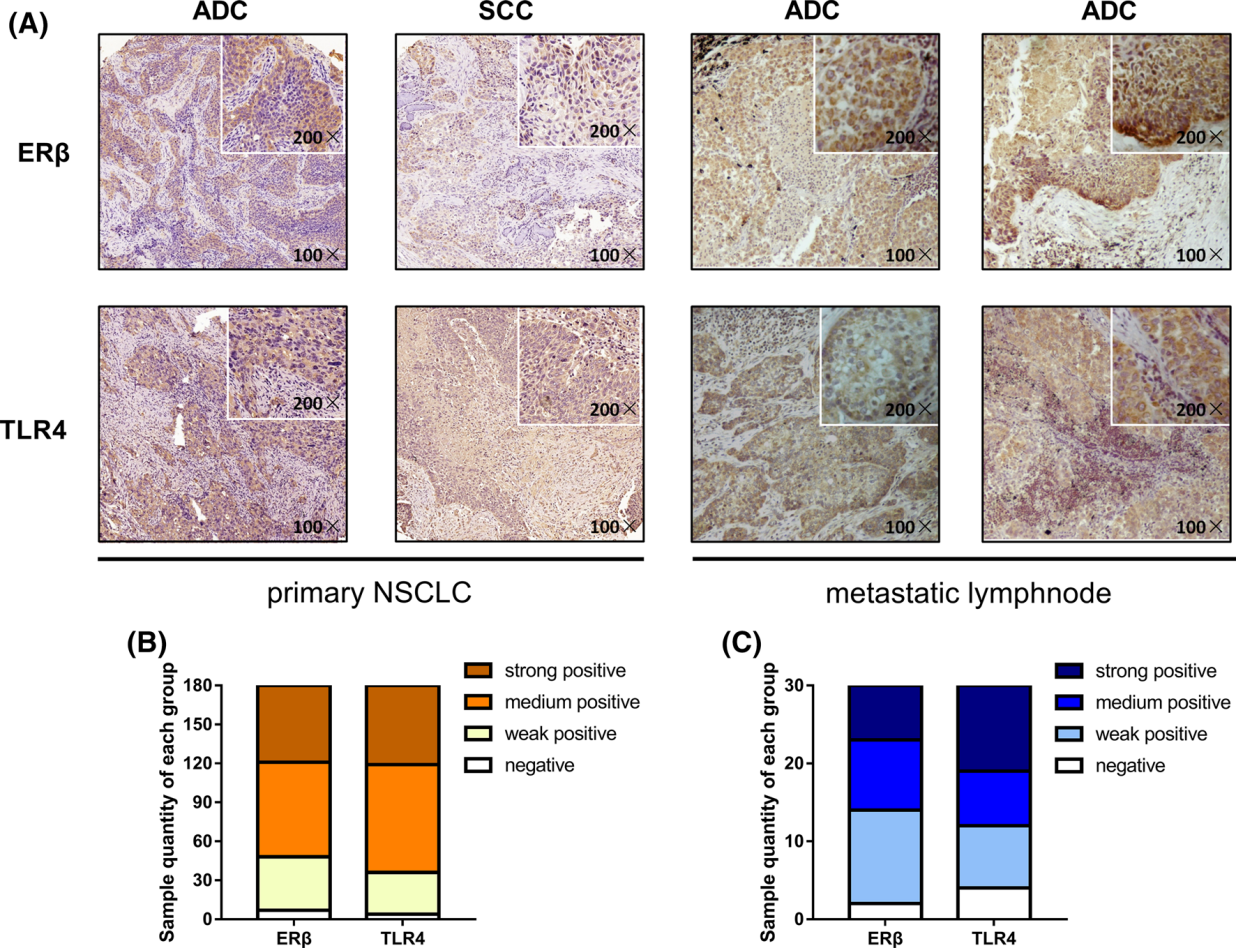
Expression of ERβ and TLR4 evaluated via immunohistochemical analyses of primary NSCLC tissue and metastatic lymph nodes. **a** Immunohistochemical analysis of ERβ and TLR4 protein expression in NSCLC tissue microarrays (TMA). NSCLC specimens were immunostained with ERβ- and TLR4-specific antibodies. Positive cells appear as a yellowish-brown color or contain yellowish-brown granules [magnifications of ×100 (large) and ×200 (small)]. **b** Histogram of the percentages of samples with different IHC staining intensities for ERβ and TLR4 (negative, weakly positive, moderately positive, strongly positive) in primary NSCLC. **c** Histogram of the percentages of samples with different IHC staining intensities for ERβ and TLR4 (negative, weakly positive, moderately positive, strongly positive) in NSCLC metastatic lymph nodes

Compared to benign pulmonary tissue, cancerous lung tissue has been shown to overexpress ERβ and TLR4 in our previous works [27, 28] and recent studies [29, 30]; however, the relation between these molecules remains unknown. To determine whether there is an association between ERβ and TLR4, we first analyzed a range of standard clinicopathological parameters and protein expression levels by immunohistochemistry (Fig. 1a). However, within our 180 samples of NSCLC tissue, no significant correlations were detected for the clinicopathological parameters, including age, sex, smoking index, histological type and clinical stage, with the expression of ERβ or TLR4 (Table 1). According to the Allred score calculated via Spearman’s analyses, a significant correlation between ERβ and TLR4 was observed (Spearman’s Rho correlation coefficient = 0.411, *p* < 0.001; Table 2). Similar percentages of different IHC staining intensities for ERβ and TLR4 are shown in Fig. 1b: strongly positive ERβ (32.78%) vs. TLR4 (33.89%) expression, moderately positive ERβ (40.55%) vs. TLR4 (46.11%) expression, weakly positive ERβ (22.78%) vs. TLR4 (17.78%) expression and negative ERβ (3.89%) vs. TLR4 (2.22%) expression. The total positive rate of ERβ expression in the primary NSCLC samples was 73.33%, and TLR4 expression appeared in 80.00%.

**Table 2:** Spearman’s correlation rate between ERβ and TLR4 expression in primary non-small cell lung carcinoma and metastatic lymphnode

	*N*	ERβ expression		Positive rate (%)	TLR4 expression		Positive rate (%)	Spearman’s correlation	*p* value
		+	−		+	−			
Primary NSCLC tumor	180	132	48	73.33	144	36	80.00	0.411	< 0.001
Metastatic lymphnode	48	40	8	83.33	37	11	77.08	0.374	0.009
Total	228	172	56		181	47			

In addition, the association between ERβ and TLR4 expression was analyzed in the corresponding 30 samples of NSCLC metastatic lymph nodes. The results showed another strong correlation in the metastatic lymph nodes via Spearman’s analyses (Spearman’s Rho correlation coefficient = 0.374, *p* = 0.009; Table 2). The positive ERβ expression rate was slightly higher in the lymph nodes (83.33%), while the TLR4-positive rate (77.08%) in the lymph nodes was lower than that in the primary tissue. The distribution of the IHC scores of the metastatic lymph nodes among the different groups was strongly positive ERβ (23.33%) vs. TLR4 (36.67%) expression, moderately positive ERβ (30.00%) vs. TLR4 (23.33%) expression, weakly positive ERβ (40.00%) vs. TLR4 (26.67%) expression and negative ERβ (6.67%) vs. TLR4 (13.33%) expression (Fig. 1c). Altogether, these data indicate coexpression of ERβ and TLR4 in the progression of NSCLC metastasis.

### The combination of fulvestrant and CLI-095 synergistically inhibited the ERβ and TLR4/myd88/MMP2 pathways

**Fig. 2:**
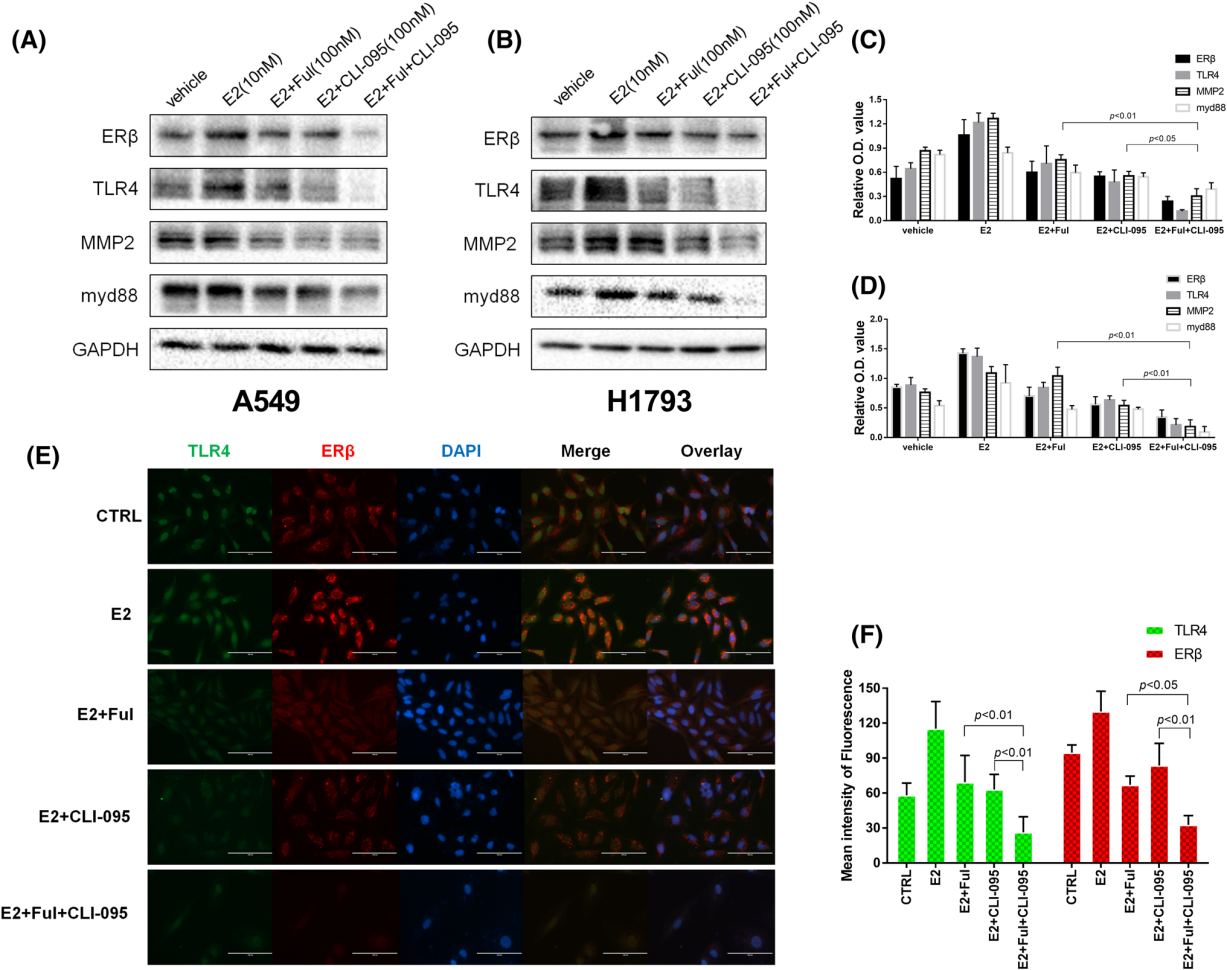
Protein expression of ERβ, TLR4 and the downstream molecules myd88 and MMP2 in NSCLC cell lines treated with estrogen and inhibitors. **a**, **b** Western blot analysis of ERβ, TLR4, myd88 and MMP2 protein levels in A549 (**a**) and H1793 (**b**) cells treated with DMSO (CTRL), E2 (10 nM), E2 + Ful (100 nM), E2 + CLI-095 (100 nM), or E2 + Ful + CLI-095 for 48 h. The combination of fulvestrant and CLI-095 synergistically inhibited the ERβ and TLR4/myd88/MMP2 pathway. **c**, **d** Detection of the relative O.D. values of the Western blot analysis. **e** Immunofluorescence staining of ERβ and TLR4 in A549 cells. Scale bar, 200 μm. **f** Detection of the mean intensity of fluorescence staining

Estrogens are known to be physiological agonists of ERβ, and the upregulation of ERβ expression, which is triggered by estrogen, has been proven to contribute to lung cancer development and metastasis [2, 31]. To determine whether the antiestrogen effect of the ER antagonist fulvestrant can be enhanced by CLI-095 or vice versa, we treated the NSCLC cell lines A549 and H1793 in different groups with E2, E2 and fulvestrant, E2 and CLI-095 or a combination of the drugs. Western blotting analysis first showed marked decreases in the levels of ERβ, TLR4 and the downstream signaling pathway molecules myd88 and MMP2 in the fulvestrant alone and CLI-095 alone groups compared to the E2 alone group. Furthermore, the lowest expression levels of each protein listed above were observed in the combination of fulvestrant and CLI-095 group (Fig. 2a–d). To support our findings, immunofluorescence analysis was used to evaluate the expression of ERβ and TLR4 in A549 cells. The strongest inhibition of protein expression was observed in the drug combination group (Fig. 2e–f). Taken together, these results indicate that fulvestrant and CLI-095 may synergistically inhibit the expression of ERβ and TLR4 as well as downstream signaling, which further enhances the antiestrogen effect.

### The inhibitory effects of fulvestrant on NSCLC cell migration and invasion were enhanced by CLI-095

**Fig. 3:**
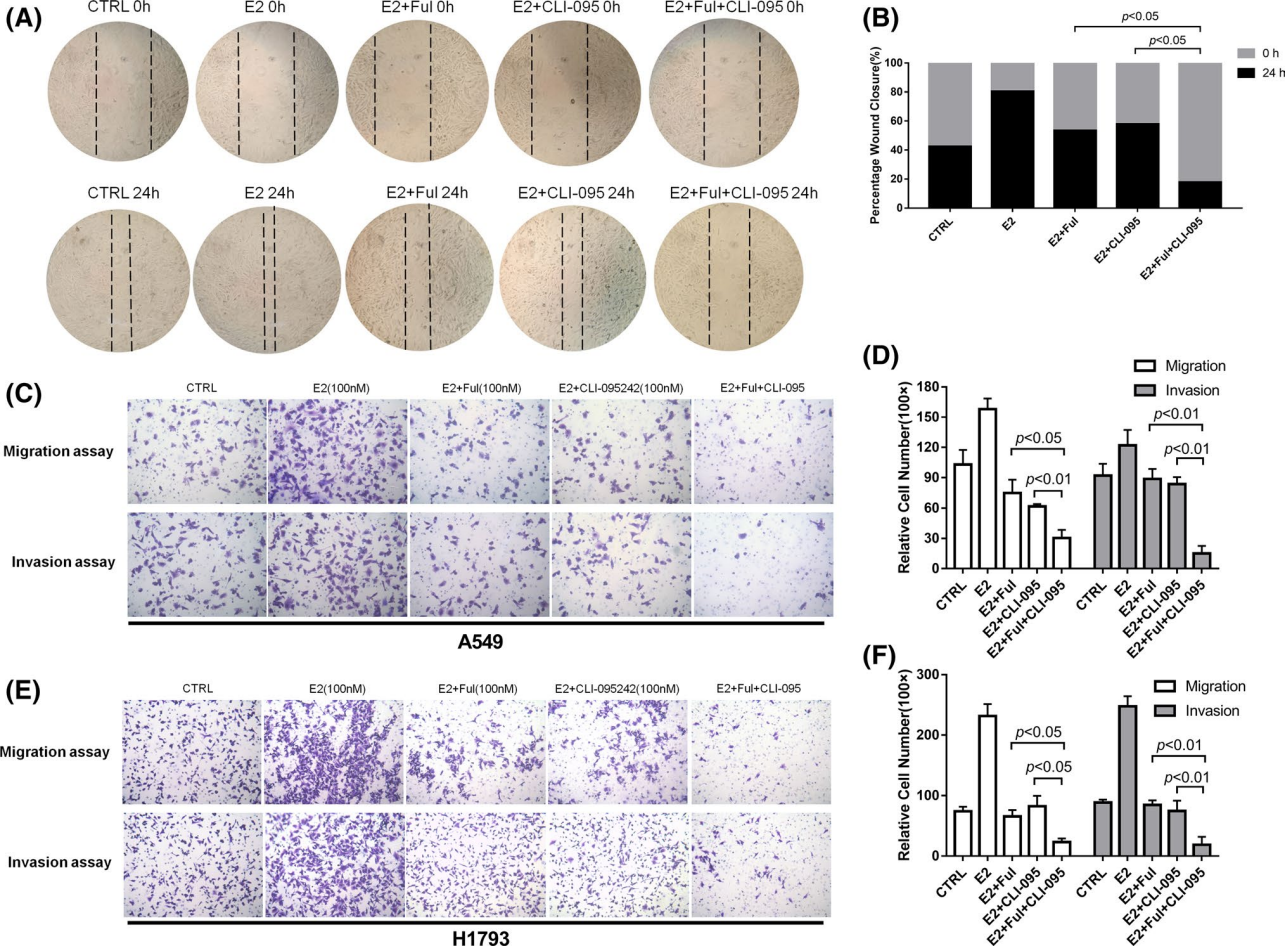
Combination of fulvestrant and CLI-095 synergistically inhibited the migratory/invasive abilities of NSCLC cells. **a** Migration of A549 cells in a wound-healing assay after treatment with DMSO (CTRL), E2 (10 nM), E2 + Ful (100 nM), E2 + CLI-095 (100 nM), or E2 + Ful + CLI-095 for 24 h (magnification ×40). **b** The effect on wound closure (shown as a percentage) by A549 cells. **c** Representative images of Transwell^®^ migration assays and Matrigel^®^‑coated Transwell^®^ invasion assays of A549 cells in different treatment groups. CLI-095 significantly enhanced the inhibitory effect of fulvestrant on the migratory/invasive abilities of NSCLC cells. **d** The counting results of the relative Transwell cell numbers in the migration and invasion assays. **e** Representative images of Transwell^®^ migration assays and Matrigel^®^‑coated Transwell^®^ invasion assays of H1793 cells in different treatment groups. CLI-095 significantly enhanced the inhibitory effect of fulvestrant on the migratory/invasive abilities of NSCLC cells. **f** The counting results of the relative Transwell cell numbers in the migration and invasion assays

To evaluate the effects of fulvestrant and CLI-095 on NSCLC cell migration and invasion, a wound-healing assay was performed. As shown in Fig. 3a, compared to E2 alone, fulvestrant and CLI-095 can both obviously inhibit the rate of lateral cell migration into a wound introduced in a confluent cellular monolayer. As expected, the combined use of fulvestrant + CLI-095 showed the strongest suppressive effect in the wound-healing assay (*p* < 0.05, Fig. 3b). Moreover, we detected and quantified the aggressiveness of NSCLC cells using Transwell^®^ migration assays and Matrigel^®^‑coated Transwell^®^ invasion assays. Similar results demonstrated that compared with treatment with E2 alone, treatment with fulvestrant or CLI-095 individually significantly inhibited the invasive ability of NSCLC cells. However, when fulvestrant and CLI-095 were combined, the results showed the lowest number of invaded NSCLC cells A549 (Fig. 3c, d) and H1793(Fig. 3e, f). Our data indicate that the addition of CLI-095 enhanced the inhibitory effects of fulvestrant on NSCLC cell migration and invasion.

### Invadopodia formation was suppressed by the fulvestrant-CLI-095 combination in NSCLC cells

**Fig. 4:**
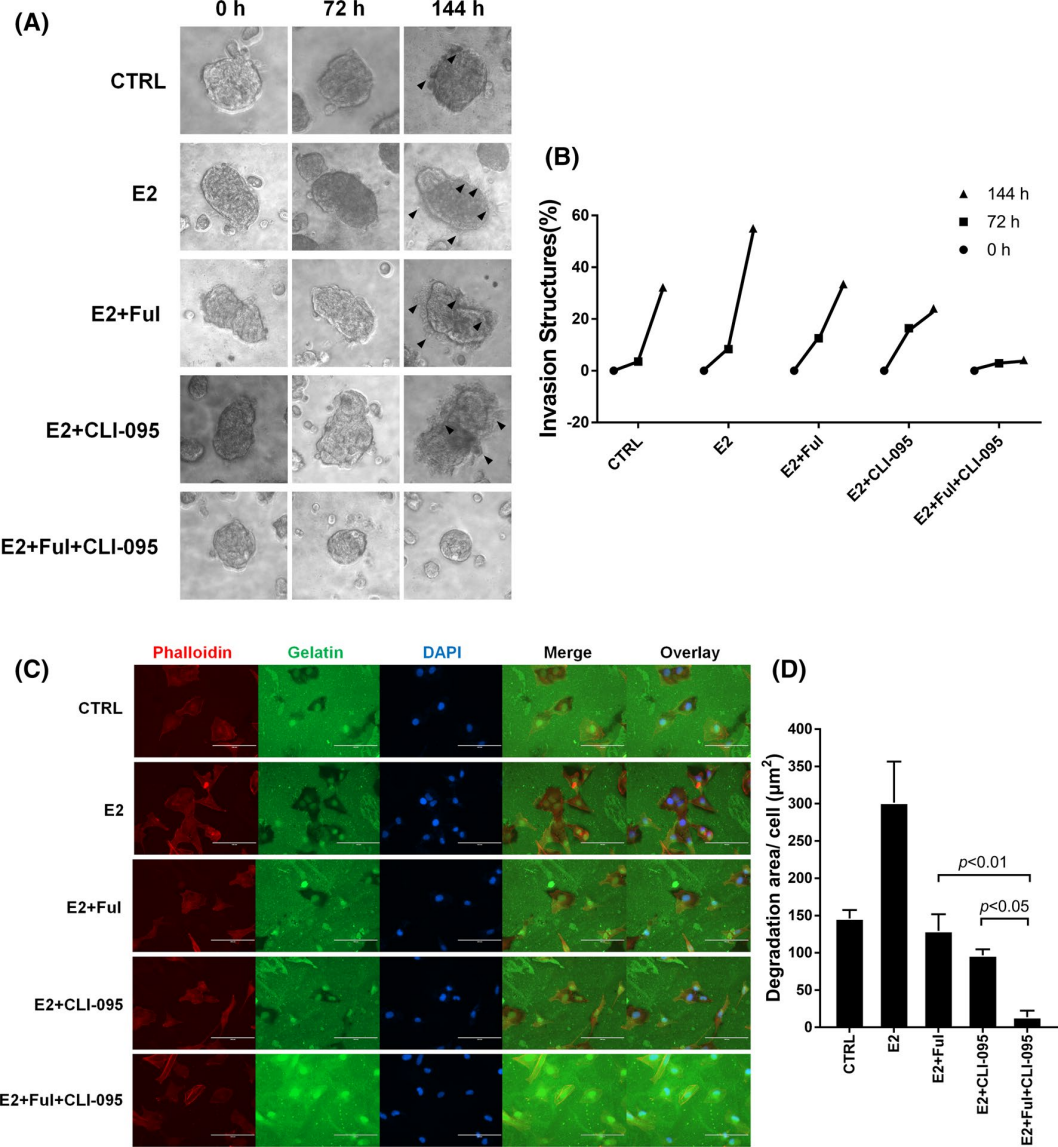
Combination treatment with Ful + CLI-095 restricted invadopodia formation in NSCLC cells. **a** 3D spheroid cell invasion assay of lung adenocarcinoma A549 cells treated with DMSO (CTRL), E2 (10 nM), E2 + Ful (100 nM), E2 + CLI-095 (100 nM), or E2 + Ful + CLI-095. Representative images were acquired at 0 h, 72 h, and 144 h (days 1, 3, and 6) via light microscopy (×100). Combination treatment with Ful + CLI-095 restricted invadopodia formation (black arrow) more than other treatments. **b** Quantification of 3D spheroid cell invasion assays. Quantification was carried out by measuring the distance between the invasive cell frontier and spheroid edge. **c** Fluorescent gelatin degradation assay. To confirm the invasive activity of cancer cells, slides were coated with FITC-conjugated gelatin (green). NSCLC cells were cultured on the FITC-conjugated gelatin-coated coverslips for 36 h. To visualize F-actin, phalloidin (red) and DAPI (blue) were used to stain the cytoplasm and nuclei, respectively. Punctuate black areas indicate representative degraded gelatin regions. Scale bar, 200 μm. **d** Quantification of the degradation level of the FITC-conjugated gelatin as assessed by the degradation assay (area per cell, μm^2^)

During metastasis, NSCLC cells invade the extracellular matrix (ECM) by forming special membrane structures called invadopodia, which can mediate the focal degradation of the pericellular ECM through the localized proteolytic activity of matrix metalloproteinases (MMPs) [32, 33]. Given the advantages provided by the 3D spheroid invasion assay, we dynamically and visually evaluated the different effects of fulvestrant and CLI-095 alone or in combination on the inhibition of the formation of invadopodia in NSCLC cells. As shown in Fig. 4a, b, E2 significantly increased the invasive capability of cells, which formed invadopodia at 144 h (6 days), compared with that of negative control cells. In contrast, fulvestrant or CLI-095 mainly slowed the formation of invadopodia in the range of 72~144 h (3–6 days). Furthermore, when ERβ and TLR4 were inhibited in combination, the formation of the invasion structures was suppressed in the range of 0~72 h (0–3 days), and the length of the invadopodia was ultimately strongly decreased.

### The combination of inhibiting ERβ and TLR4 decreased the invadopodia function and cell invasion

To fully demonstrate whether the combination of fulvestrant + CLI-095 decreases the function of invadopodia, we performed a fluorescent gelatin degradation assay in which functional aggressiveness-associated invadopodia appear as accumulations of F-actin associated with dark areas of fluorescent gelatin degradation [34, 35]. The results revealed that NSCLC cells induced with estrogen produced more invadopodia and larger areas of gelatin degradation than NSCLC cells treated with the vehicle. However, the promotive effects of estrogen on invadopodia formation and function were suppressed by fulvestrant. In addition, as shown in Fig. 4c, d, A549 cells treated with fulvestrant + CLI-095 showed an average of less than 50 μm^2^ dark degradation area/cell (*p* < 0.05), while cells treated with a single drug degraded over 100 μm^2^ area/cell. All these data make it clear that the combination of fulvestrant + CLI-095 decreased not only the formation but also the function of invadopodia and cell invasion.

## Discussion

Estrogen has recently been shown to be a key promoter in lung cancer development and metastatic progression [1, 2, 6]. Therefore, estrogen and its receptors, mainly ERβ, have the potential to become prognostic indicators and therapeutic targets in lung cancer [7, 10, 11]. Similar to hormone replacement therapy clinical trials [6], our previous work found a marked upregulation of MMP2 expression with increased aggressiveness of NSCLC cells induced by estrogen and the activation of ERβ [13]. On the other hand, MMP2, which plays roles in degrading the ECM and promoting cell invasion, is also an important component of TLR4-driven cancer metastasis [24]. Accumulating evidence in vitro and in vivo supports the fact that the activation of TLR4 and the downstream myd88/NF-κB/MMP2 axis via signaling pathways contribute to NSCLC metastasis [21, 23]. The fact that the mechanism by which TLR4 promotes metastasis is similar to that involving ERβ raises the question of whether a combination therapy targeting both TLR4 and ERβ would show stronger suppression than the use of only a single drug. The answer would help us understand the interaction between ERβ and TLR4 in tumor progression more clearly and provide evidence for a new therapeutic strategy for clinical patients suffering from advanced metastatic NSCLC.

Our results demonstrate the coexpression of ERβ and TLR4 in tissue samples from primary NSCLC tumors and the associated metastatic lymph nodes. To our knowledge, this is the first time that the expression levels of ERβ and TLR4 have been analyzed in lung cancer tissues. However, the fact that ERβ is able to regulate the TLR signaling pathways was previously demonstrated in the physiological immune system [36]. In dendritic cells and macrophages, multiple studies have shown that ER promotes the production of proinflammatory cytokines in response to TLR ligand stimulation [37]. A downregulation of TLR4 signaling and decreased secretion of IL-6 and IL-1β have been observed when a ER allele gene is conditionally deleted in myeloid cells [38]. The results above suggest a molecular basis for the interaction between ERβ and TLR4 in human cells. Consistent with our results, E2 stimulation not only led to upregulated ERβ expression in NSCLC cells but also to increased TLR4 and downstream myd88 expression. Furthermore, the combination of fulvestrant and CLI-095 showed the strongest inhibitory effect on ERβ-mediated suppression, an effect that was stronger than that of fulvestrant alone, which suggests an enhanced inhibitory effect on ERβ signaling by fulvestrant when used in conjunction with a TLR4-specific inhibitor.

We selected fulvestrant for use in these studies, because it is generally regarded as a pure antiestrogen that specifically and selectively inhibits ER signaling in target lung cancer cells [39, 40]. The antiestrogen inhibitor fulvestrant, as mentioned before, exerts antiproliferative and antimetastatic effects on NSCLC, which have been researched in vitro and in vivo in multiple studies, including our previous research [13–15, 41]. Consistent with the findings of those studies, our results clearly showed that compared with estrogen alone, fulvestrant depressed NSCLC cell migration and invasion and retarded invadopodia formation. Additionally, no significant difference was found between the vehicle and E2 + fulvestrant groups. Therefore, to further enhance the antimetastatic effect of fulvestrant, we combined CLI-095 with fulvestrant in our study. Furthermore, fulvestrant has also been repeatedly added to phase II clinical studies, which mainly aimed to enhance the therapeutic effect of an EGFR-TKI or relieve drug resistance [17, 18]. However, no significant difference in the progression-free survival rate was detected in these clinical studies, and the clinical effect of monotherapy with an antiestrogen drug remains unknown. In this respect, our results suggested that combined targeting of both ERβ and TLR4 by fulvestrant + CLI-095 may become an efficient way to enhance the clinical effect of antiestrogen therapies. Nevertheless, the indications of fulvestrant will be enlarged by future research, which will help us to understand the mechanism of antiestrogen therapy in NSCLC cells.

In contrast to the effects of targeting fulvestrant, the effects of targeting TLR4 signaling, which is specifically inhibited by CLI-095 (TAK242), have been proven to be significantly effective in inhibiting or blocking cell invasion and metastasis in many malignant tumors, such as ovarian cancer, breast cancer and glioma [42, 43]. However, research on CLI-095 remains limited to in vitro studies in NSCLC. Currently, the effect of inflammation on tumorigenesis and progression has been widely noted. Research using immunohistochemistry and enzyme-linked immunosorbent assays showed a poor prognosis in TLR4- and programmed cell death ligand 1 (PD-L1)-expressing NSCLC patients [29]. Increasing numbers of hepatic metastasis nodules of NSCLC cells have been observed in a mouse model treated with LPS [21]. Furthermore, evidence suggests that knocking down TLR4 expression by siRNA significantly suppresses the constitutive phosphorylation of Akt and PI3K, and this knockdown may contribute to the inhibition of tumor growth [26]. Although recent studies have provided multiple lines of evidence that the activation of TLR4 and its downstream signaling pathways significantly promotes NSCLC development and metastasis, none of these studies selected CLI-095 to block TLR4 signaling. Our results are the first to prove that CLI-095 treatment can significantly inhibit TLR4 downstream signaling; additionally, our results showed that treatment with CLI-095 suppressed the enhancement of aggressiveness in NSCLC cells induced by estrogen and enhanced the antitumor effect of fulvestrant. The results above may further suggest the possibility that there is an interaction between ERβ and TLR4 at the molecular level.

## Conclusion

In conclusion, our data provide comprehensive insight into the roles of ERβ and TLR4 in NSCLC. The inhibition of ERβ and TLR4 signaling significantly suppressed NSCLC cell migration and invasion and retarded invadopodia formation. Combining the targeting of ERβ by the selective inhibitor fulvestrant with the targeting of TLR4 by CLI-095 may be a potential therapeutic strategy for patients suffering from advanced NSCLC. We plan to further investigate the detailed mechanism underlying the roles of ERβ and TLR4 in NSCLC cells, with a focus on the further development of antiestrogen therapy for NSCLC treatment.

## Electronic supplementary material

Below is the link to the electronic supplementary material.

Supplementary file1 (TIF 559 kb) (A) Combination of Fulvestrant and CLI-095 inhibited cell proliferation stimulated by E2 in NSCLC cell lines. Cell Counting Kit-8 assay of lung adenocarcinoma A549 cells treated with DMSO (CTRL), E2 (10 nM), E2+Ful (100 nM), E2+CLI-095 (100 nM), or E2+Ful+CLI-095. The optical density (OD) value proportional to the cell number was measured and plotted on the growth curve. (B) Dose-response curves of NSCLC cell lines A549 and H1793 to Fulvestrant and CLI-095. Cell invasion rate was determined by transwell invasion assay in the presence of various doses of drugs

## Data Availability

The datasets generated and analyzed in the current study are available from the corresponding author upon reasonable request.

## Abbreviations

NSCLC: Non-small cell lung cancer
ERβ: Estrogen receptor beta
TLR4: Toll-like receptor 4
MMP2: Matrix Metalloproteases 2
IHC: Immunohistochemical
LPS: Lipopolysaccharide
E2: Estrogen
Ful: Fulvestrant
HRT: Hormone replacement
WHI: Women’s Health Initiative
TMA: Tissue microarray
myd88: Myeloid differentiation factor 88
NF-κB: Nuclear factor κB
