# Supplementary figures and images for: Targeting Toll-like receptor 4 with CLI-095 (TAK-242) enhances the antimetastatic effect of the estrogen receptor antagonist fulvestrant on non-small cell lung cancer
Source: Clin Transl Oncol. 2020 May 4;22(11):2074–86. doi: 10.1007/s12094-020-02353-3 (PMC7505887; doi:10.1007/s12094-020-02353-3)

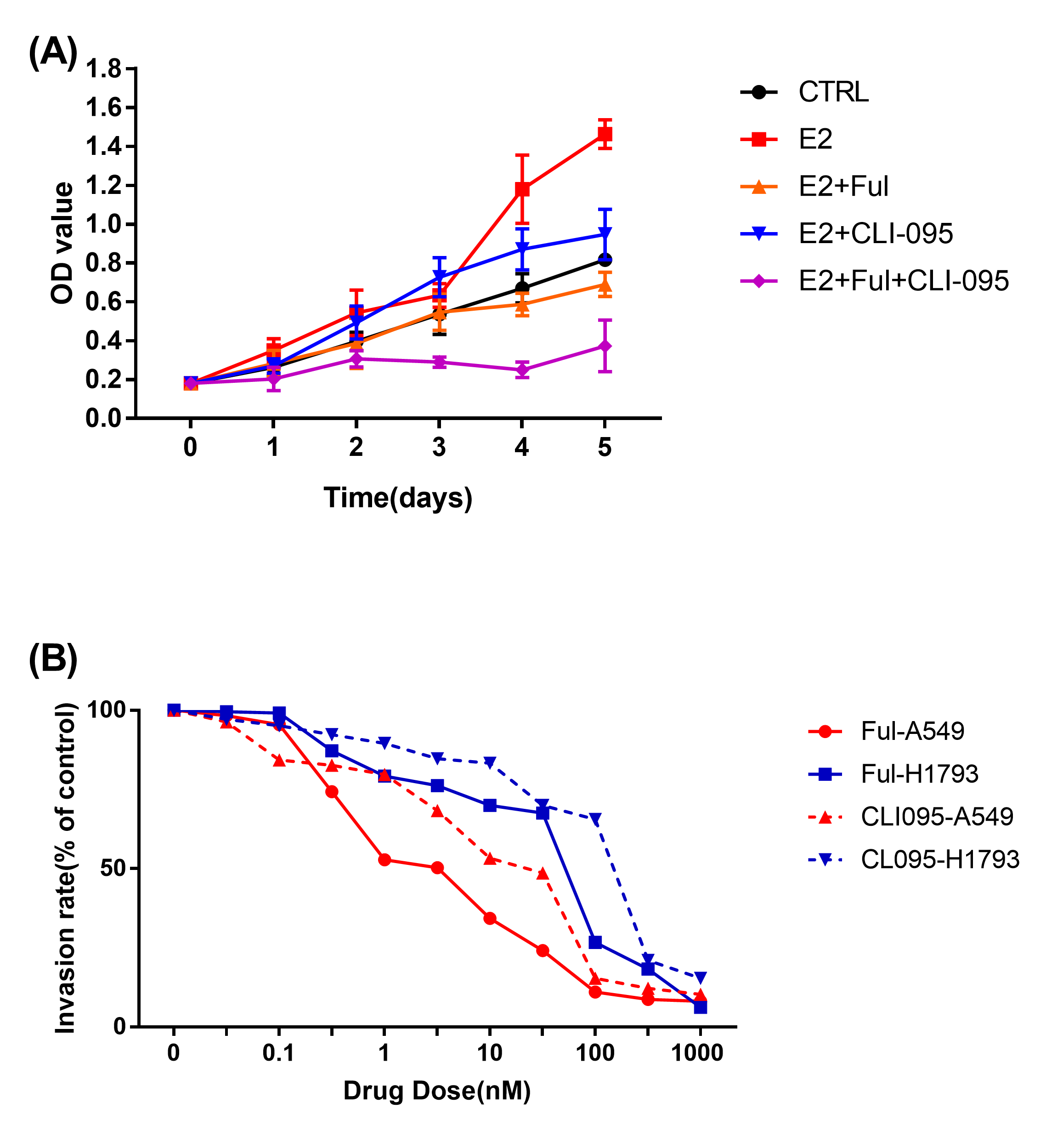

Supplement: Supplementary file 1 — Supplementary file1 (TIF 559 kb) (A) Combination of Fulvestrant and CLI-095 inhibited cell proliferation stimulated by E2 in NSCLC cell lines. Cell Counting Kit-8 assay of lung adenocarcinoma A549 cells treated with DMSO (CTRL), E2 (10 nM), E2+Ful (100 nM), E2+CLI-095 (100 nM), or E2+Ful+CLI-095. The optical density (OD) value proportional to the cell number was measured and plotted on the growth curve. (B) Dose-response curves of NSCLC cell lines A549 and H1793 to Fulvestrant and CLI-095. Cell invasion rate was determined by transwell invasion assay in the presence of various doses of drugs [file 12094_2020_2353_MOESM1_ESM.tif]
